# Effects of the COVID-19 pandemic on mental health, anxiety, and depression

**DOI:** 10.1186/s40359-023-01130-5

**Published:** 2023-04-11

**Authors:** Ida Kupcova, Lubos Danisovic, Martin Klein, Stefan Harsanyi

**Affiliations:** 1 Institute of Medical Biology, Genetics and Clinical Genetics, Faculty of Medicine, Comenius University in Bratislava, Sasinkova 4, Bratislava, 811 08 Slovakia; 2 Institute of Histology and Embryology, Faculty of Medicine, Comenius University in Bratislava, Sasinkova 4, Bratislava, 811 08 Slovakia

**Keywords:** COVID-19, Pandemic, Mental health, Anxiety, Depression, Slovakia

## Abstract

**Background:**

The COVID-19 pandemic affected everyone around the globe. Depending on the country, there have been different restrictive epidemiologic measures and also different long-term repercussions. Morbidity and mortality of COVID-19 affected the mental state of every human being. However, social separation and isolation due to the restrictive measures considerably increased this impact. According to the World Health Organization (WHO), anxiety and depression prevalence increased by 25% globally. In this study, we aimed to examine the lasting effects of the COVID-19 pandemic on the general population.

**Methods:**

A cross-sectional study using an anonymous online-based 45-question online survey was conducted at Comenius University in Bratislava. The questionnaire comprised five general questions and two assessment tools the Zung Self-Rating Anxiety Scale (SAS) and the Zung Self-Rating Depression Scale (SDS). The results of the Self-Rating Scales were statistically examined in association with sex, age, and level of education.

**Results:**

A total of 205 anonymous subjects participated in this study, and no responses were excluded. In the study group, 78 (38.05%) participants were male, and 127 (61.69%) were female. A higher tendency to anxiety was exhibited by female participants (p = 0.012) and the age group under 30 years of age (p = 0.042). The level of education has been identified as a significant factor for changes in mental state, as participants with higher levels of education tended to be in a worse mental state (p = 0.006).

**Conclusions:**

Summarizing two years of the COVID-19 pandemic, the mental state of people with higher levels of education tended to feel worse, while females and younger adults felt more anxiety.

## Introduction

The first mention of the novel coronavirus came in 2019, when this variant was discovered in the city of Wuhan, China, and became the first ever documented coronavirus pandemic [1–3]. At this time there was only a sliver of fear rising all over the globe. However, in March 2020, after the declaration of a global pandemic by the World Health Organization (WHO), the situation changed dramatically [4]. Answering this, yet an unknown threat thrust many countries into a psycho-socio-economic whirlwind [5, 6]. Various measures taken by governments to control the spread of the virus presented the worldwide population with a series of new challenges to which it had to adjust [7, 8]. Lockdowns, closed schools, losing employment or businesses, and rising deaths not only in nursing homes came to be a new reality [9–11]. Lack of scientific information on the novel coronavirus and its effects on the human body, its fast spread, the absence of effective causal treatment, and the restrictions which harmed people´s social life, financial situation and other areas of everyday life lead to long-term living conditions with increased stress levels and low predictability over which people had little control [12].

Risks of changes in the mental state of the population came mainly from external risk factors, including prolonged lockdowns, social isolation, inadequate or misinterpreted information, loss of income, and acute relationship with the rising death toll. According to the World Health Organization (WHO), since the outbreak of the COVID-19 pandemic, anxiety and depression prevalence increased by 25% globally [13]. Unemployment specifically has been proven to be also a predictor of suicidal behavior [14–18]. These risk factors then interact with individual psychological factors leading to psychopathologies such as threat appraisal, attentional bias to threat stimuli over neutral stimuli, avoidance, fear learning, impaired safety learning, impaired fear extinction due to habituation, intolerance of uncertainty, and psychological inflexibility. The threat responses are mediated by the limbic system and insula and mitigated by the pre-frontal cortex, which has also been reported in neuroimaging studies, with reduced insula thickness corresponding to more severe anxiety and amygdala volume correlated to anhedonia as a symptom of depression [19–23]. Speaking in psychological terms, the pandemic disturbed our core belief, that we are safe in our communities, cities, countries, or even the world. The lost sense of agency and confidence regarding our future diminished the sense of worth, identity, and meaningfulness of our lives and eroded security-enhancing relationships [24].

Slovakia introduced harsh public health measures in the first wave of the pandemic, but relaxed these measures during the summer, accompanied by a failure to develop effective find, test, trace, isolate and support systems. Due to this, the country experienced a steep growth in new COVID-19 cases in September 2020, which lead to the erosion of public´s trust in the government´s management of the situation [25]. As a means to control the second wave of the pandemic, the Slovak government decided to perform nationwide antigen testing over two weekends in November 2020, which was internationally perceived as a very controversial step, moreover, it failed to prevent further lockdowns [26]. In addition, there was a sharp rise in the unemployment rate since 2020, which continued until July 2020, when it gradually eased [27]. Pre-pandemic, every 9th citizen of Slovakia suffered from a mental health disorder, according to National Statistics Office in 2017, the majority being affective and anxiety disorders. A group of authors created a web questionnaire aimed at psychiatrists, psychologists, and their patients after the first wave of the COVID-19 pandemic in Slovakia. The results showed that 86.6% of respondents perceived the pathological effect of the pandemic on their mental status, 54.1% of whom were already treated for affective or anxiety disorders [28].

In this study, we aimed to examine the lasting effects of the COVID-19 pandemic on the general population. This study aimed to assess the symptoms of anxiety and depression in the general public of Slovakia. After the end of epidemiologic restrictive measures (from March to May 2022), we introduced an anonymous online questionnaire using adapted versions of Zung Self-Rating Anxiety Scale (SAS) and Zung Self-Rating Depression Scale (SDS) [29, 30]. We focused on the general public because only a portion of people who experience psychological distress seek professional help. We sought to establish, whether during the pandemic the population showed a tendency to adapt to the situation or whether the anxiety and depression symptoms tended to be present even after months of better epidemiologic situation, vaccine availability, and studies putting its effects under review [31–34].

## Materials and Methods

**Fig. 1:**
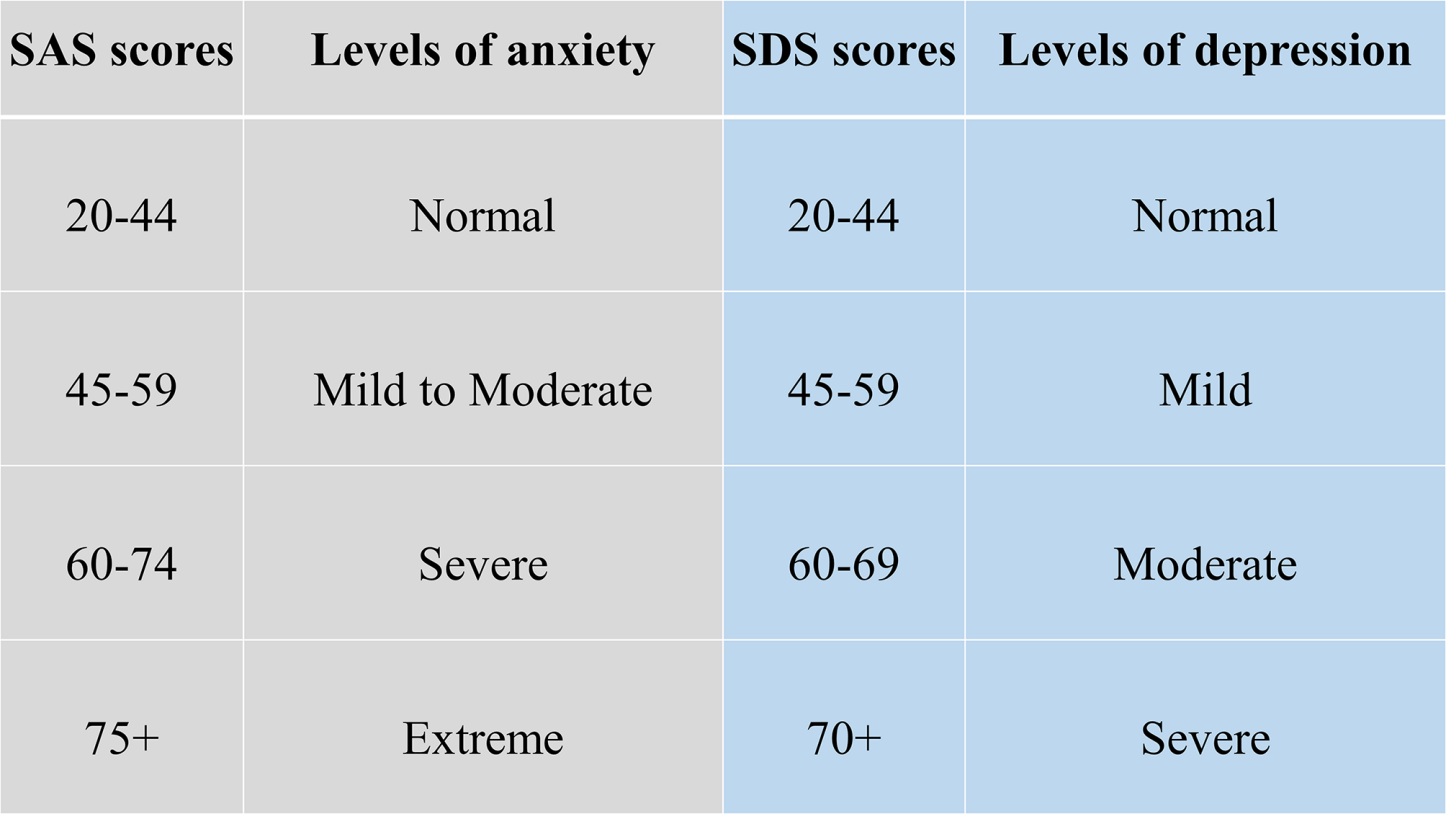
Categories of Zung SAS and SDS scores with clinical interpretation

This study utilized a voluntary and anonymous online self-administered questionnaire, where the collected data cannot be linked to a specific respondent. This study did not process any personal data. The questionnaire consisted of 45 questions. The first three were open-ended questions about participants’ sex, age (date of birth was not recorded), and education. Followed by 2 questions aimed at mental health and changes in the will to live. Further 20 and 20 questions consisted of the Zung SAS and Zung SDS, respectively. Every question in SAS and SDS is scored from 1 to 4 points on a Likert-style scale. The scoring system is introduced in Fig. 1. Questions were presented in the Slovak language, with emphasis on maintaining test integrity, so, if possible, literal translations were made from English to Slovak. The questionnaire was created and designed in Google Forms®. Data collection was carried out from March 2022 to May 2022. The study was aimed at the general population of Slovakia in times of difficult epidemiologic and social situations due to the high prevalence and incidence of COVID-19 cases during lockdowns and social distancing measures. Because of the character of this web-based study, the optimal distribution of respondents could not be achieved.

During the course of this study, 205 respondents answered the anonymous questionnaire in full and were included in the study. All respondents were over 18 years of age. The data was later exported from Google Forms® as an Excel spreadsheet. Coding and analysis were carried out using IBM SPSS Statistics version 26 (IBM SPSS Statistics for Windows, Version 26.0, Armonk, NY, USA). Subject groups were created based on sex, age, and education level. First, sex due to differences in emotional expression. Second, age was a risk factor due to perceived stress and fear of the disease. Last, education due to different approaches to information. In these groups four factors were studied: (1) changes in mental state; (2) affected will to live, or frequent thoughts about death; (3) result of SAS; (4) result of SDS. For SAS, no subject in the study group scored anxiety levels of “severe” or “extreme”. Similarly for SDS, no subject depression levels reached “moderate” or “severe”. Pearson’s chi-squared test(χ2) was used to analyze the association between the subject groups and studied factors. The results were considered significant if the p-value was less than 0.05.

Ethical permission was obtained from the local ethics committee (Reference number: ULBGaKG-02/2022). This study was performed in line with the principles of the Declaration of Helsinki. All methods were carried out following the institutional guidelines. Due to the anonymous design of the study and by the institutional requirements, written informed consent for participation was not required for this study.

## Results

In the study, out of 205 subjects in the study group, 127 (62%) were female and 78 (38%) were male. The average age in the study group was 35.78 years of age (range 19–71 years), with a median of 34 years. In the age group under 30 years of age were 34 (16.6%) subjects, while 162 (79%) were in the range from 31 to 49 and 9 (0.4%) were over 50 years old. 48 (23.4%) participants achieved an education level of lower or higher secondary and 157 (76.6%) finished university or higher. All answers of study participants were included in the study, nothing was excluded.

In Tables 1 and 2, we can see the distribution of changes in mental state and will to live as stated in the questionnaire. In Table 1 we can see a disproportion in education level and mental state, where participants with higher education tended to feel worse much more than those with lower levels of education. Changes based on sex and age did not show any statistically significant results.

**Table 1:** Distribution of changes in mental state in the study group during the coronavirus pandemic depending on sex, age, and education

Variables		Change in mental state			χ2 p-Value
		I feel worse	No change	I feel better	
Sex	Male	37	35	6	0.169
	Female	77	44	6	
Age	< 30	22	12	-	0.139
	31–49	90	61	11	
	> 50	2	6	1	
Education	Lower or Higher Secondary	17	27	4	0.006
	University or higher	97	52	8	

In Table 2. we can see, that decreased will to live and frequent thoughts about death were only marginally present in the study group, which suggests that coping mechanisms play a huge role in adaptation to such events (e.g. the global pandemic). There is also a possibility that living in times of better epidemiologic situations makes people more likely to forget about the bad past.

**Table 2:** Distribution of affected will to live and thoughts about death in the study group during the coronavirus pandemic depending on sex, age, and education

Variables		Affected will live, thoughts about death				χ2 p-Value
		Never	Sometimes	Often	Almost always	
Sex	Male	61	11	4	2	0.860
	Female	100	14	8	5	
Age	< 30	25	5	2	2	0.818
	31–49	128	20	9	5	
	> 50	8	-	1	-	
Education	Lower or Higher Secondary	38	7	2	1	0.819
	University or higher	123	18	10	6	

Anxiety and depression levels as seen in Tables 3 and 4 were different, where female participants and the age group under 30 years of age tended to feel more anxiety than other groups. No significant changes in depression levels based on sex, age, and education were found.

**Table 3:** Distribution of anxiety levels in the study group during the coronavirus pandemic depending on sex, age, and education

Variables		Zung’s anxiety scale		χ2 p-Value
		Normal range	Mild to Moderate Levels	
Sex	Male	70	8	0.012
	Female	96	31	
Age	< 30	23	11	0.042
	31–49	134	28	
	> 50	9	-	
Education	Lower or Higher Secondary	40	8	0.634
	University or higher	126	31	

**Table 4:** Distribution of depression levels in the study group during the coronavirus pandemic depended on sex, age, and education

Variables		Zung’s depression scale		χ2 p-Value
		Normal Range	Mildly Depressed	
Sex	Male	29	49	0.149
	Female	35	92	
Age	< 30	10	24	0.272
	31–49	49	113	
	> 50	5	4	
Education	Lower or Higher Secondary	17	31	0.473
	University or higher	47	110	

## Discussion

Compared to the estimated global prevalence of depression in 2017 (3.44%), in 2021 it was approximately 7 times higher (25%) [14]. Our study did not prove an increase in depression, while anxiety levels and changes in the mental state did prove elevated. No significant changes in depression levels go in hand with the unaffected will to live and infrequent thoughts about death, which were important findings, that did not supplement our primary hypothesis that the fear of death caused by COVID-19 or accompanying infections would enhance personal distress and depression, leading to decreases in studied factors. These results are drawn from our limited sample size and uneven demographic distribution. Suicide ideations rose from 5% pre-pandemic to 10.81% during the pandemic [35]. In our study, 9.3% of participants experienced thoughts about death and since we did not specifically ask if they thought about suicide, our results only partially correlate with suicidal ideations. However, as these subjects exhibited only moderate levels of anxiety and mild levels of depression, the rise of suicide ideations seems unlikely. The rise in suicidal ideations seemed to be especially true for the general population with no pre-existing psychiatric conditions in the first months of the pandemic [36]. The policies implemented by countries to contain the pandemic also took a toll on the population´s mental health, as it was reported, that more stringent policies, mainly the social distancing and perceived government´s handling of the pandemic, were related to worse psychological outcomes [37]. The effects of lockdowns are far-fetched and the increases in mental health challenges, well-being, and quality of life will require a long time to be understood, as Onyeaka et al. conclude [10]. These effects are not unforeseen, as the global population suffered from life-altering changes in the structure and accessibility of education or healthcare, fluctuations in prices and food insecurity, as well as the inevitable depression of the global economy [38].

The loneliness associated with enforced social distancing leads to an increase in depression, anxiety, and posttraumatic stress in children in adolescents, with possible long-term sequelae [39]. The increase in adolescent self-injury was 27.6% during the pandemic [40]. Similar findings were described in the middle-aged and elderly population, in which both depression and anxiety prevalence rose at the beginning of the pandemic, during the pandemic, with depression persisting later in the pandemic, while the anxiety-related disorders tended to subside [41]. Medical professionals represented another specific at-risk group, with reported anxiety and depression rates of 24.94% and 24.83% respectively [42]. The dynamic of psychopathology related to the COVID-19 pandemic is not clear, with studies reporting a return to normal later in 2020, while others describe increased distress later in the pandemic [20, 43].

Concerning the general population, authors from Spain reported that lockdowns and COVID-19 were associated with depression and anxiety [44]. In January 2022 Zhao et al., reported an elevation in hoarding behavior due to fear of COVID-19, while this process was moderated by education and income levels, however, less in the general population if compared to students [45]. Higher education levels and better access to information could improve persons’ fear of the unknown, however, this fact was not consistent with our expectations in this study, as participants with university education tended to feel worse than participants with lower education. A study on adolescents and their perceived stress in the Czech Republic concluded that girls are more affected by lockdowns. The strongest predictor was loneliness, while having someone to talk to, scored the lowest [46]. Garbóczy et al. reported elevated perceived stress levels and health anxiety in 1289 Hungarian and international students, also affected by disengagement from home and inadequate coping strategies [47]. Wathelet et al. conducted a study on French University students confined during the pandemic with alarming results of a high prevalence of mental health issues in the study group [48]. Our study indicated similar results, as participants in the age group under 30 years of age tended to feel more anxious than others.

In conclusion, we can say that this pandemic changed the lives of many. Many of us, our family members, friends, and colleagues, experienced life-altering events and complicated situations unseen for decades. Our decisions and actions fueled the progress in medicine, while they also continue to impact society on all levels. The long-term effects on adolescents are yet to be seen, while effects of pain, fear, and isolation on the general population are already presenting themselves.

The limitations of this study were numerous and as this was a web-based study, the optimal distribution of respondents could not be achieved, due to the snowball sampling strategy. The main limitation was the small sample size and uneven demographic distribution of respondents, which could impact the representativeness of the studied population and increase the margin of error. Similarly, the limited number of older participants could significantly impact the reported results, as age was an important risk factor and thus an important stressor. The questionnaire omitted the presence of COVID-19-unrelated life-changing events or stressors, and also did not account for any preexisting condition or risk factor that may have affected the outcome of the used assessment scales.

## Data Availability

The datasets generated and analyzed during the current study are not publicly available due to compliance with institutional guidelines but they are available from the corresponding author (SH) on a reasonable request.
